# Cross-ecosystem subsidies drive CO_2_ flux rates in a coastal ecotone

**DOI:** 10.1371/journal.pone.0357828

**Published:** 2026-09-11

**Authors:** Kyle A. Emery, Thomas Lenihan, Nicholas K. Schooler, Robert J. Miller, Henry M. Page, Donna M. Schroeder, Stephen G. Whitaker, David M. Hubbard, Jenifer E. Dugan

**Affiliations:** 1 Marine Science Institute, University of California, Santa Barbara, California, United States of America; 2 Department of Ecology, Evolution and Marine Biology, University of California, Santa Barbara, California, United States of America; 3 Bureau of Ocean Energy Management, Pacific OCS Region, Camarillo, California, United States of America; 4 Channel Islands National Park, U.S. National Park Service, Ventura, California, United States of America; IEAPM: Instituto de Estudos do Mar Almirante Paulo Moreira, BRAZIL

## Abstract

Ecotones and the steep environmental gradients they represent are often hotspots of biodiversity and ecosystem functioning. Consumer activities can mediate ecosystem functions, including decomposition in these hotspots. For example, cross-ecosystem subsidies of marine macrophyte wrack support diverse, productive intertidal food webs on temperate sandy beaches creating hotspots of organic matter processing that are highly vulnerable to growing disturbance threats. As a proxy for wrack decomposition rates, we compared the CO_2_ flux from the biologically active high tide strandline of bare intertidal beach sands on 14 beaches of California’s Northern Channel Islands that varied greatly in the abundance of wrack and macroinvertebrate wrack consumers. Wrack cover (0.03 to 12 m^2^ m^-1^) and consumer abundance (339–112,747 individuals m^-1^) varied by three orders of magnitude across sites. Species richness, biomass, and abundance of wrack-associated macroinvertebrates increased with wrack cover. CO_2_ flux (0.05 to 1.2 g CO_2_ m^-2^ hour^-1^) was strongly correlated with wrack cover, and wrack consumers likely mediated this relationship. Estimated rates of beach-scale respiration by macroinvertebrate consumers varied over 50-fold among sites (8–449 mg CO_2_ hour^-1^ m^-1^). The estimated fraction of beach CO_2_ from wrack consumers averaged 11.8%, but ranged from 0.9 to 37.7% among sites. Wrack was the dominant driver of CO_2_ flux while consumers likely modulated this relationship. Our results demonstrate how strong coupling of subsidies, intertidal consumers, and biogeochemical processes affects a key ecosystem function on sandy beaches, a shoreline ecotone imperiled by rising sea levels and coastal development.

## Introduction

Ecotones, or transition zones between ecosystems, occupy relatively small areas yet are recognized as hotspots for biodiversity and ecosystem functioning [1,2]. However, as climate continues to change and redistribute species, these areas may be particularly vulnerable to losses of both biodiversity and ecosystem functioning [3–5]. Elimination of such hotspots may carry disproportionately greater impacts on the respective ecosystems than predicted by their spatial extent [6,7]. Hotspots of enhanced biogeochemical activity are especially prevalent at terrestrial-aquatic interfaces, creating locations of significantly higher process rates relative to the rest of the system [8,9]. Many ecosystems feature hotspots that affect nutrient cycling and transformation, including streams with patchy fish distributions [10], freshwater mussel aggregations [11], large ungulate carcasses [12], vernal pools [7], and whale falls [13,14]. Rates of function in hotspots may be enhanced by the activity of consumers responding to a subsidy, as shown for fish [10] and large carcasses like ungulates and whales [12–14]. While hotspots in an ecosystem are generally associated with functions or processes, they can also encompass community-based metrics, such as disproportionately high biodiversity [15,16], metacommunity connectivity [17], key source populations [18], community composition boundaries [19], and presence of functionally rare species [20].

The coastal ecotones and hotspots of intertidal shoreline ecosystems can have high process rates [21] yet may be particularly vulnerable to climate change because of the compounding influence of marine and terrestrial forcings [22,23]. Some coastal ecosystems are also uniquely imperiled because of their inherent dependence on resources from other ecosystems [24,25]. As shoreline ecosystems with low *in situ* primary productivity and high dependence on subsidies from other productive marine ecosystems to support their food webs and biodiversity [26–29], combined with growing human impacts, sandy beaches are particularly vulnerable to climate forcing. The high tide strandline of beaches is the principal zone of wrack subsidy deposition and retention, forms a key habitat and foraging zone for upper intertidal macroinvertebrates, shorebirds, and small mammals, and represents a hotspot of biodiversity and ecosystem functioning [27,30–33].

On sandy beaches the remineralization of stranded marine wrack to its inorganic components is facilitated by processes that include the feeding activity (e.g., shredding) and excretion of intertidal consumers (detritivores), concurrent with or followed by microbial decomposition. As macroinvertebrates consume and process wrack [28,34], nutrient remineralization is enhanced compared to wrack deposits in the absence of consumers [35]. Macroalgal wrack deposits on beaches have been recognized as biogeochemical hotspots with higher levels of sediment-to-atmosphere CO_2_ fluxes than bare sand, attributed primarily to microbial activity [36,37]. The type and amount of macroalgal wrack also affect both CO_2_ flux and nutrient remineralization rates with greater values observed in mixed species and larger wrack deposits [21,36]. The temperature dependence of these processes observed in some cases also indicates that global warming could enhance rates of CO_2_ and nutrient release from wrack deposited on beaches [38–40].

A quantitative understanding of how an ecosystem functions under current conditions is required to predict how it may respond to anthropogenic and climate change [41–43]. To address this need for sandy beaches, we used a space-for-time approach, where spatial variability is used as a proxy for the effects of change (increases or decreases) over time [44,45], to evaluate the impacts of organic matter inputs on intertidal sediment CO_2_ flux across a natural gradient in wrack standing stock and wrack consumers. We expected CO_2_ flux to scale with the abundance and species richness of intertidal macroinvertebrate wrack detritivores. To test these hypotheses, we measured CO_2_ flux and surveyed wrack cover and macroinvertebrate wrack consumer communities on 14 sandy beaches located on four of California’s Northern Channel Islands that experience little to no direct impact from human activities compared to mainland beaches. To assess the relative contribution of different intertidal macroinvertebrate wrack consumers to observed total beach-scale CO_2_ flux we used values from laboratory-measured respiration rates for seven common species scaled by their biomass as measured at each beach.

## Methods

### Study sites and survey schedule

**Fig 1:**
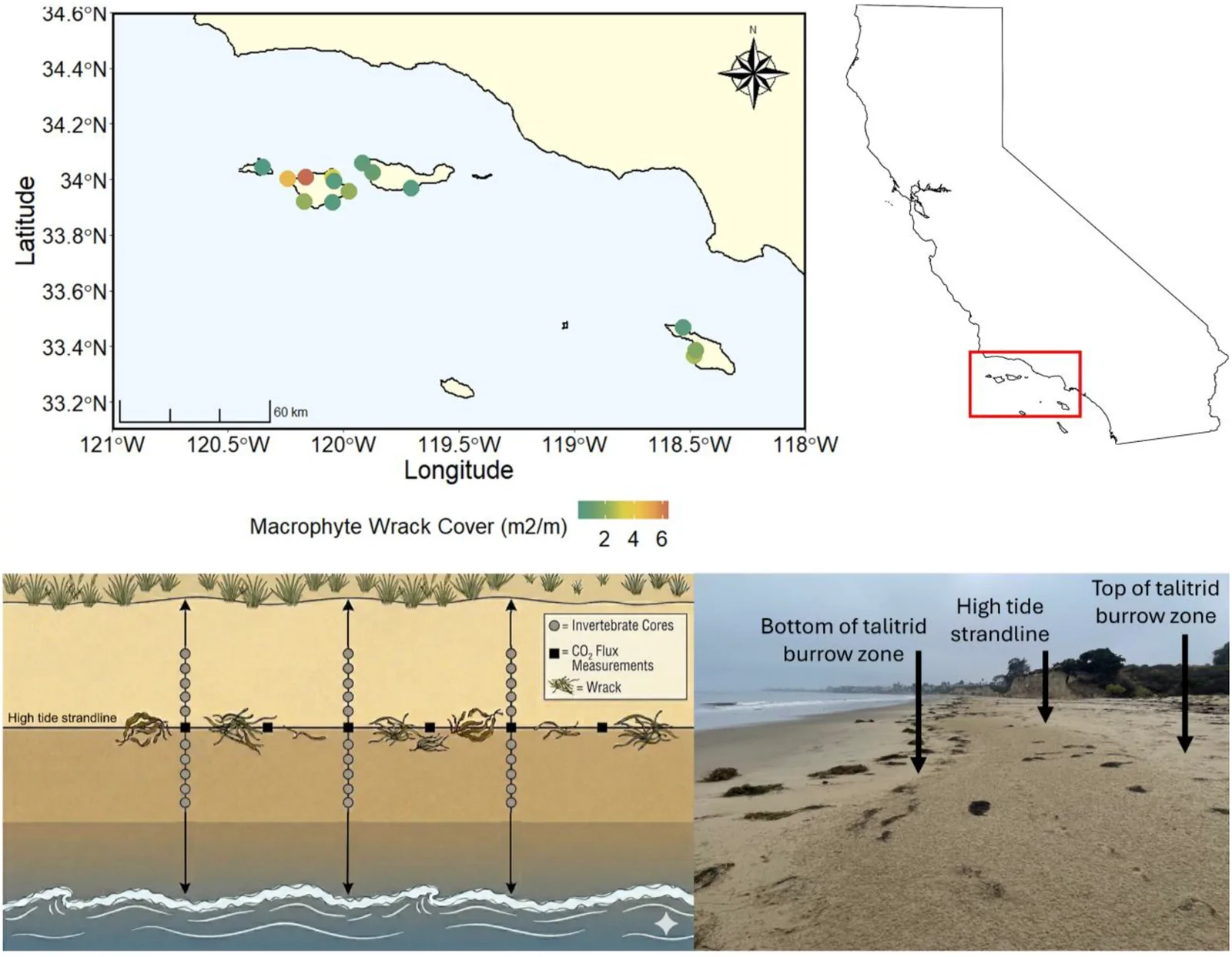
Study site locations and wrack cover. Top panel: Locations of the study beaches and their wrack cover on the California Channel Islands, California, USA. Color-scale indicates the mean cover of macrophyte wrack for each site. Islands from east to west are Santa Catalina Island, Santa Cruz Island, Santa Rosa Island, and San Miguel Island. Map images are provided by the U.S. Census Bureau and are in the public domain. Bottom Panel: A schematic of the sandy beach survey zone where grey circles represent core locations (10 cores in each of two zones collected, five shown here for display purposes). The talitrid zone, as illustrated in the photo on the right, is the more seaward set of cores. The five CO_2_ flux measurement locations are indicated with black squares in the schematic. Gemini AI was used to generate the schematic diagram.

We surveyed 14 open coast sandy beaches located on four of the California Channel Islands (Fig 1) during the summer to fall period, when maximum beach width, wrack cover, and macroinvertebrate populations are present prior to winter storm-driven beach erosion. Seasonal effects on habitat and fauna are minimal during this period. Surveys were conducted in September 2016, 2017, and 2018 for Santa Rosa Island, October 2016 and August 2017 and 2018 for Santa Cruz Island, November 2017 and October 2018 for San Miguel Island, and October 2018 for Santa Catalina Island. Beaches were sampled one to three times, depending on the site (see S1 Table in S1 File for beach characteristics from each sampling event). Sampling was conducted under a California Department of Fish and Wildlife Scientific Collecting Permit, Specific Use (PERMIT SC 726), and access to field sites and logistical support was provided by the Channel Islands National Park, United States National Park Service, the Santa Cruz Island UC Natural Reserve, The Nature Conservancy, USC Wrigley Institute for Environmental Studies, and the Catalina Island Conservancy.

### Field surveys

At each study site, we measured marine macrophyte wrack cover and estimated macroinvertebrate richness, abundance, and biomass on three shore-normal transects (five transects for Santa Rosa Island surveys) from the back-beach limit (e.g., cliff base, dunes, etc.) to the upper swash limit within two hours of low tide (Fig 1, See S1 Table in S1 File for beach characteristics). Wrack cover was measured using a line intercept method and averaged for each site visit [27]. Upper intertidal beach macroinvertebrates were sampled by taking two sets of 10 aggregated sediment cores per transect (not sampled at San Miguel Island due to unexploded ordinance risk). Ten cores were evenly spaced from the back-beach limit to the top of the talitrid amphipod burrow zone, and ten cores were evenly spaced from the top to the bottom of the talitrid amphipod burrow zone (Fig 1). The talitrid amphipod burrow zone was determined by finding the upper and lower limits of burrowed talitrids by visually identifying burrows and excavating sand around to determine where the presence of talitrids ceased (Fig 1). Of the wrack-associated intertidal invertebrate species, talitrid amphipods are generally the most abundant organism and burrow the lowest on the beach face [46]. All cores were aggregated and sieved through a 1.5 mm mesh bag in the surf zone, and the contents were transferred to 3.785 L (one-gallon) resealable bags, and frozen. In the laboratory, samples were sorted and organisms were identified to species level, counted, and weighed to the nearest mg wet weight and averaged for each survey date. Species-specific allometric relationships were used to convert wet weight to dry weight and ash-free dry weight (AFDW) for the beach invertebrate samples (S2 Table in S1 File).

The high tide strandline is biologically active (e.g., fresh wrack deposition, high macroinvertebrate abundance and richness) on the study beaches. The flux of CO_2_ from wrack in field observations is highest along the high tide strandline, where sand does not dry completely between tides [47]. Above this zone where the sand is dry, and below it where the sand is saturated, we found little to no CO_2_ flux (S1 Figure in S1 File). During each survey, we made five sediment CO_2_ flux measurements on the 24-hour high tide strandline (Fig 1) using an EGM-5 Portable CO_2_ Gas Analyzer with a dark SRC-2 Soil Respiration Chamber and white PVC collars (PP Systems, Amesbury, MA, USA). For each CO_2_ flux measurement, the chamber was flushed with air by holding it upright for 25 seconds, placed on a collar, equilibrated for 10 seconds, and then run for 180 seconds to produce an integrated linear respiration rate (g CO_2_ m^-2^ hour^-1^) under dark conditions. We calculated the mean respiration rate from the five values for each survey. Air temperature was also recorded during each run. Measurement rates represent net fluxes of CO_2_ at the sediment surface and all measurements in this study were net fluxes out of the sediment, there was no net uptake of CO_2_. To ensure consistency of measurements across surveys, all flux measurements were taken over sand with no wrack or visible animal activity within the collar. Our measurements therefore represent CO_2_ flux from wrack-influenced sediments rather than directly from wrack patches. While this may result in an underestimate of CO_2_ flux from wrack itself, it provides a more conservative estimate of high tide strandline or beach-scale fluxes.

### Laboratory measurements

To explore differences in respiration across wrack consumer species and their potential contribution to the net respiration rates measured on the study beaches we chose the most common and abundant detritivore (wrack-consuming) species from our sediment core samples for direct measurements of species-specific respiration rates in the laboratory. Our laboratory measurements do not account for indirect effects on CO_2_ fluxes, such as enhancement through bioturbation, shredding, or stimulation of microbial activity. Two isopod species (*Alloniscus perconvexus* and *Tylos punctatus*), a beetle (*Phaleria rotundata*), and four talitrid amphipods (*Megalorchestia californiana*, *M. corniculata*, *M. benedicti*, and *M. minor*) were collected on beaches in Santa Barbara County, California, USA and analyzed within 24 hours of collection. Respiration trials were run for six adult-sized individuals for each species.

Respiration rates of each species were estimated using stop-flow respirometry. Eight respirometry chambers, consisting of 50 cc plastic syringes, were connected to the system for each testing period. The system was set up with push-mode plumbing where airflow stemmed from a compressed air canister containing a mixture of gas equivalent to normal atmospheric air that was passed through mass flow controllers (Sierra Series 830 Mass Flow Controller; Sierra Instruments, Monterey, CA, USA) that maintained airflow at a constant rate of 60 mL min^-1^. While a reasonable proxy, normal atmospheric air may differ from oxygen conditions in burrows and result in different respiration rates than what may be measured using in situ gas concentrations. Two scrubber columns consisting of two layers of Drierite separated by a layer of Ascarite (II) were placed upstream from the respirometry chambers. The airflow was distributed to the chambers by way of a multiplexor, which split the airflow between each chamber (RM8 multiplexor, Sable Systems, Inc., Las Vegas, NV, U.S.A.), as well as directing another channel of air to the reference cell of a CO_2_ analyzer ((LI-7000, LI-COR, Lincoln, NE, U.S.A.). Airflow through each of the eight respirometry chambers was automatically switched by programming the multiplexor to a period of 8 minutes per chamber, except for a control chamber that was opened for 90 seconds. While each chamber received airflow, CO_2_ levels (ppm) were recorded with the LI-COR analyzer once per second. The respirometry system was flushed with atmospheric air for 10 minutes before and after each recording.

The analyzer was calibrated daily before the experiment began and displayed consistent baseline levels with no drift in the measurements. Nitrogen gas was used as a reference gas along with 100 ppm CO_2_ span gas to calibrate the analyzer to the proper measuring scale and to regularly test the instrument’s accuracy. The total volume of CO_2_ gas (ppm) in each chamber was recorded using Expedata v.1.1.18 (SSI). The respirometry chambers were cleaned after every test with a solution of isopropyl alcohol before being rinsed and dried. The temperature of the room was recorded during all testing periods using a thermistor probe placed directly outside the respirometry chambers. All respirometry trials were performed at room temperature (mean 20.62°C; 19.15–21.92°C range) under natural light conditions. Following these measurements, individuals were frozen and later measured for dry weight (drying oven, 48 hours at 60°C) and ash-free dry weight (loss on ignition, 4 hours at 500°C) to the nearest mg.

Out of the eight respirometry chambers, six were dedicated to measuring the respiration rates of individual organisms, and two were left empty every measurement period. One chamber (control-1) served as a control to ensure measured CO_2_ volumes were not changed by outside factors. The other empty respirometry chamber (control-2) provided a baseline CO_2_ level for the recording, quantifying any background levels of CO_2_ that might inflate the final respiratory rate values of individuals in the other chambers. The CO_2_ levels of the control-2 chamber were recorded for 90 seconds before and after each of the other chambers. Within each respirometry trial, the amount of CO_2_ gas (ppm) produced by each individual and the background control chamber was recorded once every 68 minutes, with the six chambers holding animals and control-1 open to gas flow for eight minutes each (56 minutes in total), and the baseline chamber (control-2) open for 90 seconds at regular intervals between each of the other chambers’ eight-minute runs (12 minutes in total).

### Data analysis

We used linear mixed-effects models fit by restricted maximum likelihood (REML) to establish the relationship between log-transformed macrophyte wrack cover and log-transformed mean macroinvertebrate abundance (individuals m^-1^) and species richness with year nested within site as the random factor for both models. Next, we explored the relationship between mean macrophyte wrack cover (m^2^ m^-1^) and beach CO_2_ flux (g CO_2_ m^2^ hour^-1^) using a linear mixed-effects model fit by REML with year nested within site as the random factor. We then compared total mean log-transformed macroinvertebrate abundance (individuals m^-1^) and species richness to beach CO_2_ flux using the same linear mixed-effects model approach fit by REML with year nested within site as the random factor. Lastly, we utilized a linear mixed-effects model framework fit by maximum likelihood with air temperature, upper beach width, wrack cover, macroinvertebrate species richness, and macroinvertebrate abundance (S1 Table in S1 File) as fixed factors and year nested within site as random factors to identify the primary drivers of CO_2_ flux [48]. We then compared this model against a null intercept model with the same random factors [49].

The raw output of CO_2_ (ppm) as measured by the LI-COR analyzer in the stop-flow respirometry setup represented the total volume (i.e., build-up) of CO_2_ gas in each chamber after a 68-minute period. The raw measures of CO_2_ (ppm) produced by each individual were converted to CO_2_ (mL) using the transformation and macro utility tools in Expedata v.1.1.18 (SSI). The rate of CO_2_ produced per minute (VCO_2_; mL CO_2_ min^-1^), a proxy for respiration rate, was then calculated by taking the area (the volume of CO_2_) under each CO_2_ curve measured for each eight-minute window of time in which the CO_2_ output of each individual was measured. The volumes of CO_2_ gas recorded in the animal chambers and control-1 were then standardized by averaging the 90-second control (control-2) chamber values to obtain a common baseline for each 68-minute testing interval. This baseline was applied to each of the integrated VCO_2_ curves using the macro utility tool in Expedata v.1.1.18 (SSI). The first two hours of respiration rate measurements were discarded to allow for animal acclimation to the measurement system. Rate measurements, calculated from the baseline-corrected VCO_2_ (mL CO 2 min^-1^) values from the third hour, were normalized by individual biomass (AFDW) and averaged for the six replicates per species to produce mean species-specific biomass-normalized respiration rates. Respiration rates of all talitrid amphipods were aggregated to develop a genus-level equation for determining respiration rate from AFDW biomass and respiration rates of all seven tested species were aggregated to develop a community-level equation for biomass-derived respiration rate. We used OLS linear regression to develop a model of log-transformed respiration rate (mg CO_2_ hour^-1^ individual^-1^) against log-transformed ash-free dry weight (mg) for *Megalorchestia* and for the full suite of seven species.

Next, we scaled our laboratory-based species respiration rate measurements to whole-beach rates based on the biomass of the seven detritivore species using a Monte Carlo error propagation approach. For each species, beach scale estimates of biomass (AFDW mg m^-1^) were multiplied by the respective respiration rates (mg CO_2_ hour^-1^ AFDW mg^-1^) to obtain estimates of total detritivore respiration (mg CO_2_ hour^-1^ m^-1^). This was done by approximating the mean and standard deviation of both metrics through 10,000 simulations across a normal distribution, bounded by 0, for every species and site combination. The reported mean and standard deviations for each species and site combination is the mean and standard deviation fo the simulated totals. To place these values into an ecosystem-level context, we estimated the proportion of our beach scale measurements of CO_2_ flux that can be attributed to these seven highly abundant detritivore species. As most of the beach CO_2_ flux occurs in the talitrid amphipod zone along the high tide strandline (S1 Figure in S1 File), we scaled our CO_2_ measurements by multiplying the flux rate by the width of this zone for each beach. Then, we estimated the proportion of this flux that could be attributed to the consumer species by dividing the beach-scale CO_2_ flux rate by the estimated consumer CO_2_ flux rate. All analyses were conducted in R [50,51].

## Results

**Fig 2:**
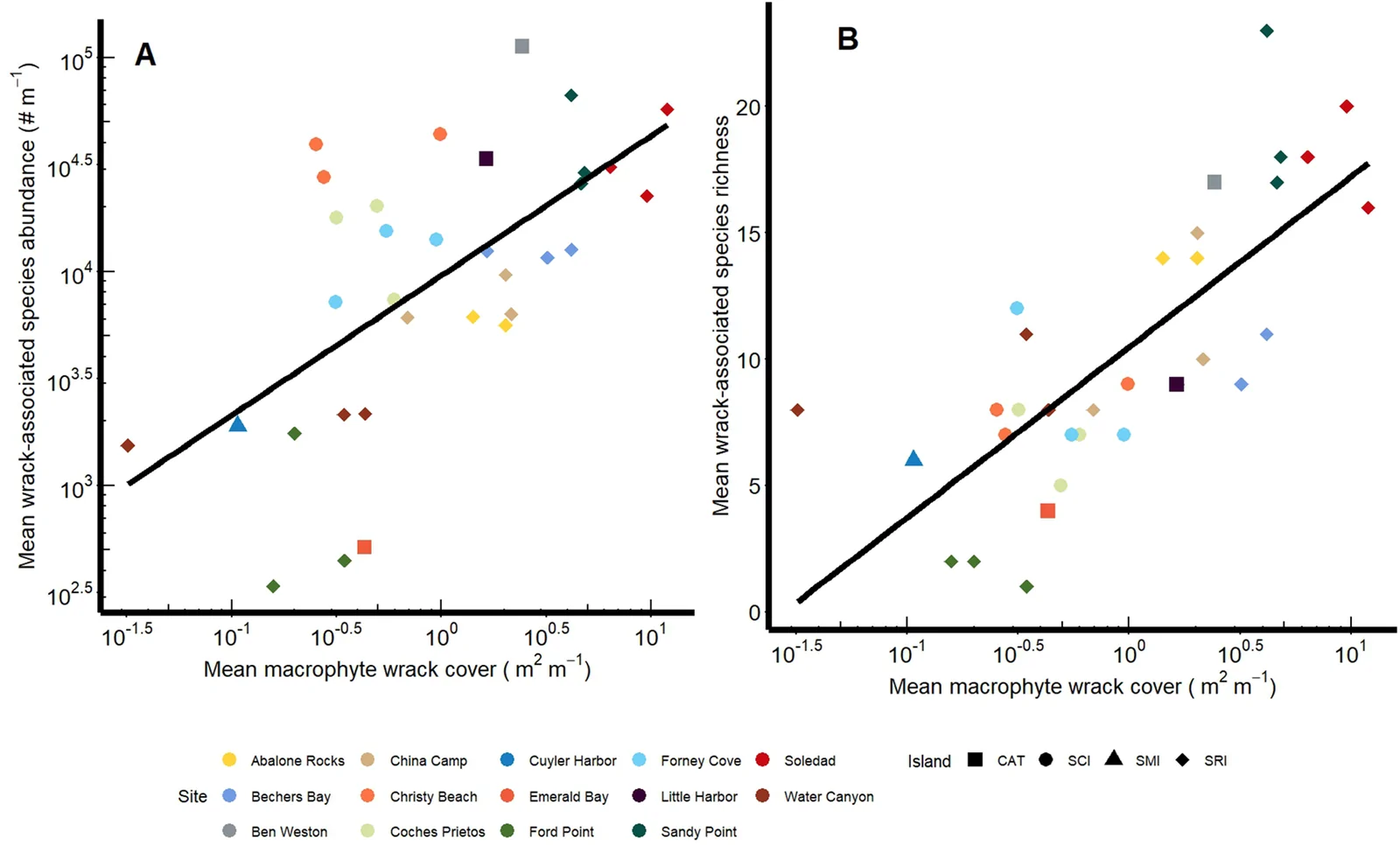
Wrack and the macroinvertebrate community. The relationship between a) log-transformed upper beach macroinvertebrate mean abundance (Slope = 0.31, SE = 0.014, t = 2.2, p = 0.04) and b) mean species richness (Slope = 4.7, SE = 1.3, t = 3.7, p = 0.002) to log-transformed mean macrophyte wrack cover. Data point shape indicates the study island and data point color indicates the study beach. Data points are mean values for a site visit from 2016 to 2018 (year not graphically displayed and sites visited 1 to 3 times).

The standing stock of marine macrophyte wrack on beaches was primarily comprised of giant kelp (*Macrocystis pyrifera*, 48.6%) and surfgrass (*Phyllospadix spp.*, 44.6%). Wrack abundance or standing stock (as cover) varied three orders of magnitude across all beaches and surveys (0.03 to 12.0 m^2^ m^-1^, Table 1 and Fig 1). Peak values of wrack abundance (>3 m^2^ m^-1^) were observed at three beaches on Santa Rosa Island (Table 1 and Fig 1). Similarly, the abundance and biomass of wrack-associated intertidal macroinvertebrates ranged across three (339–112,747 individuals m^-1^) and two (10.4 to 2247.7 g m^-1^) orders of magnitude, respectively, among sites and surveys. The total species richness of wrack-associated macroinvertebrates varied from 1 to 23 species among sites and surveys. Wrack cover and the log-transformed mean abundance (Fig 2A, Slope = 0.31, SE = 0.014, t = 2.2, p = 0.04) and the mean species richness (Fig 2B, Slope = 4.7, SE = 1.3, t = 3.7, p = 0.002) of wrack-associated macroinvertebrates were tightly coupled across island beaches.

**Table 1:** Mean values ± standard deviation (SD) of CO_2_ flux and marine wrack cover for the surveys conducted at each study site for survey dates. CAT = Santa Catalina Island, SCI = Santa Cruz Island, SRI = Santa Rosa Island, SMI = San Miguel Island.

Island	Site	Year	Marine wrack cover (m^2^ m^-1^)	Marine wrack cover SD	CO_2_ Flux (g CO_2_ m^-2^ hour^-1^)	CO_2_ Flux SD
CAT	Ben Weston	2018	2.43	0.64	0.14	0.03
	Little Harbor	2018	1.64	0.60	0.21	0.09
	Emerald Bay	2018	0.43	0.43	0.10	0.04
SCI	Coches	2016	0.49	0.32	0.08	0.01
		2017	0.60	0.20	0.08	0.01
		2018	0.32	0.14	0.12	0.03
	Christy’s	2016	0.28	0.27	0.08	0.02
		2017	0.25	0.13	0.13	0.05
		2018	0.99	0.56	0.22	0.11
	Forney’s	2016	0.31	0.23	0.15	0.05
		2017	0.95	0.59	0.13	0.02
		2018	0.55	0.24	0.26	0.04
SRI	Abalone Rocks	2016	1.42	0.63	0.20	0.11
		2017	2.03	0.79	0.13	0.06
	Water Canyon	2016	0.43	0.49	0.06	0.01
		2017	0.03	0.02	0.05	0.03
		2018	0.34	0.32	0.09	0.04
	Bechers	2016	1.65	1.48	0.34	0.26
		2017	4.18	2.33	0.22	0.11
		2018	3.21	0.62	0.46	0.23
	Ford Pt	2016	0.20	0.40	0.13	0.05
		2017	0.16	0.23	0.17	0.03
		2018	0.35	0.12	0.18	0.06
	China Camp	2016	2.16	1.59	0.23	0.13
		2017	0.69	0.42	0.23	0.10
		2018	2.03	0.81	0.09	0.02
	Soledad	2016	11.99	3.04	0.41	0.15
		2017	9.59	1.43	1.18	1.49
		2018	6.39	1.64	0.67	0.27
	Sandy Pt	2016	4.18	1.78	0.38	0.12
		2017	4.84	2.54	0.38	0.05
		2018	4.64	2.58	0.46	0.20
SMI	Cuyler’s	2017	0.11	0.10	0.07	0.01
		2018	0.07	0.04	0.10	0.01

**Fig 3:**
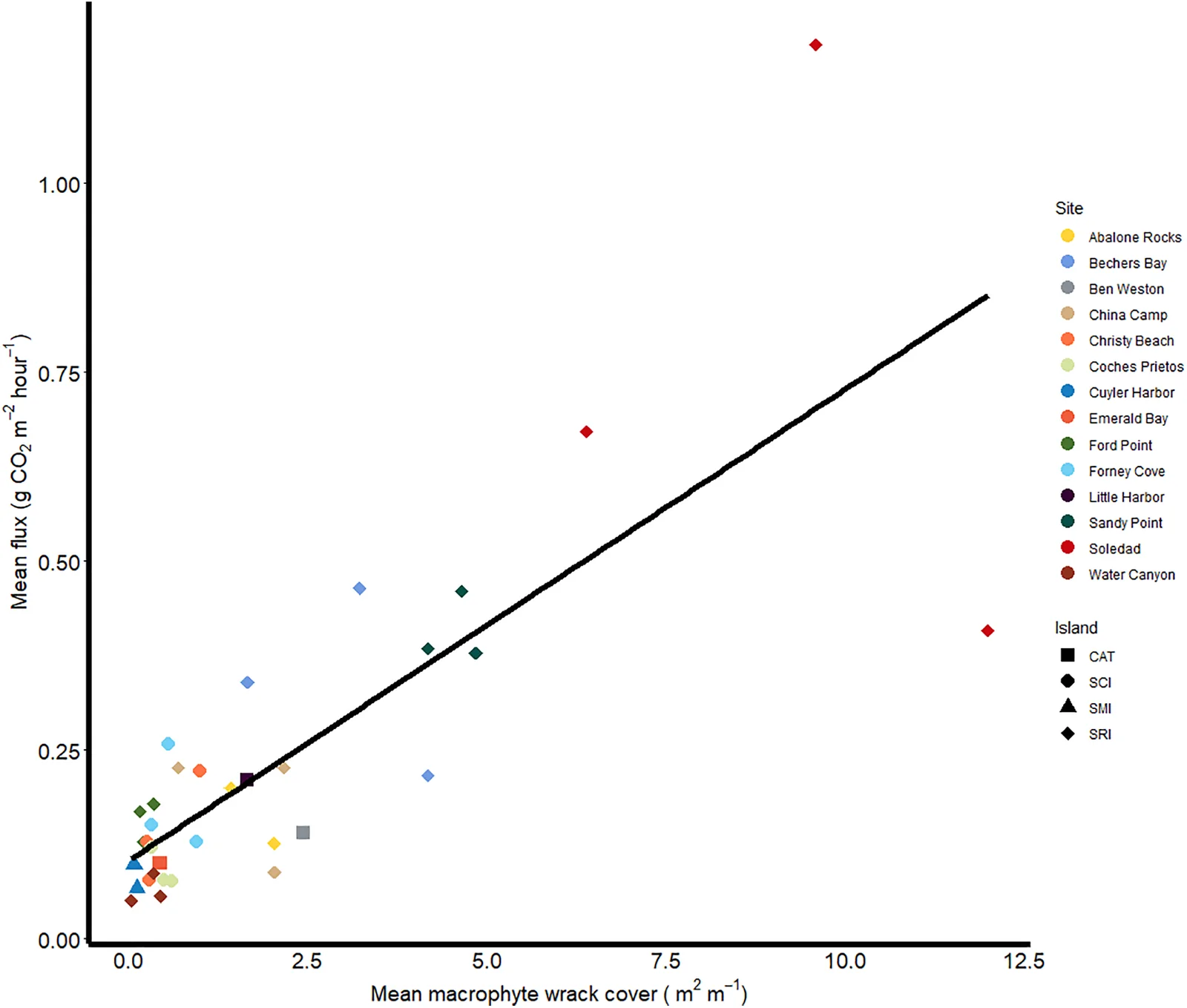
Wrack and CO_2_ flux. The relationship between mean values of CO_2_ flux (g CO_2_ m^-2^ hour^-1^) and cover of macrophyte wrack (Slope = 0.06, SE = 0.009, t = 7.09, p < 0.0001). Data point shape indicates the study island and data point color indicates the study beach. Data points are mean values for a site visit from 2016 to 2018 (year not graphically displayed and sites visited 1 to 3 times).

**Fig 4:**
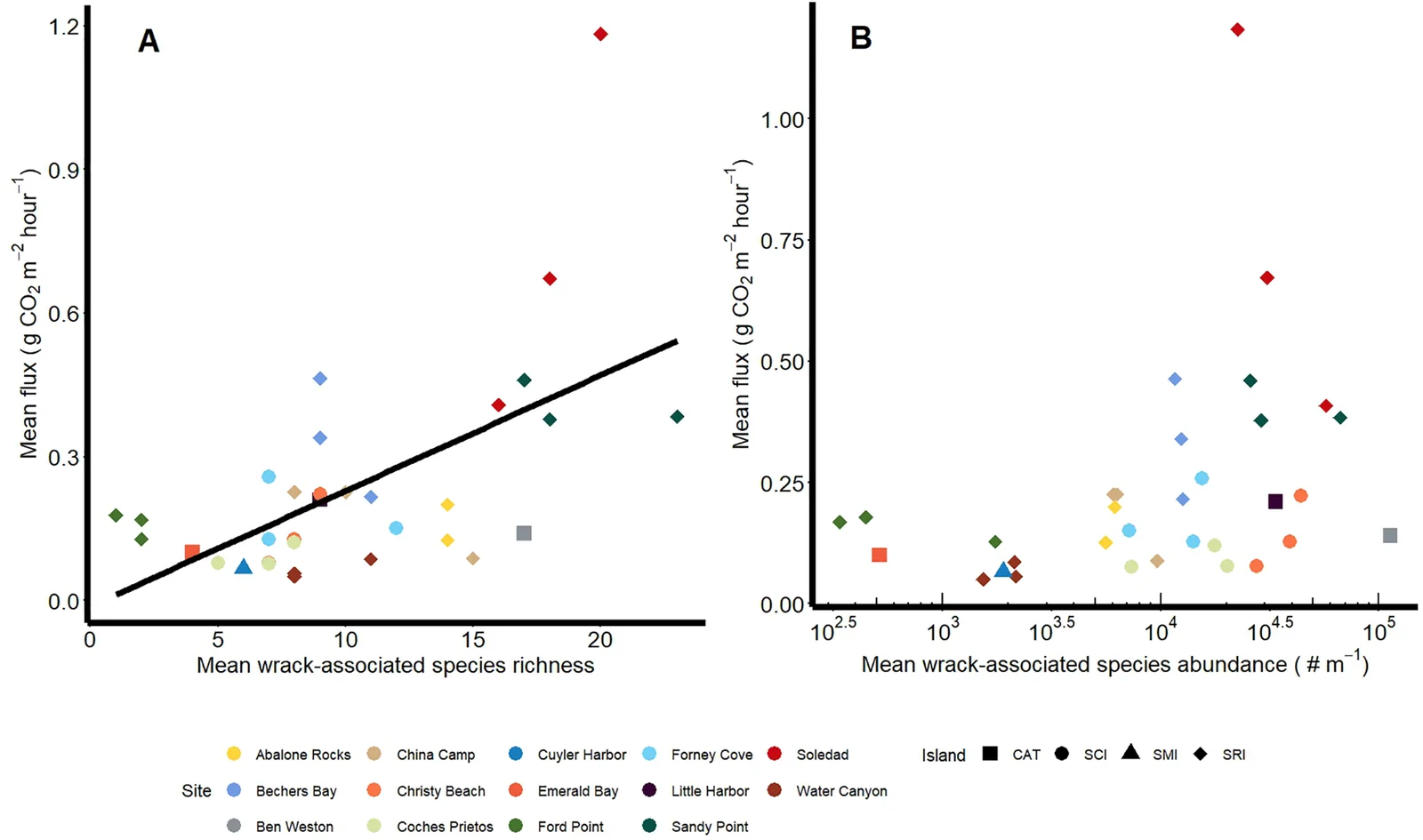
CO_2_ flux and the macroinvertebrate community. The relationship between upper beach wrack-associated macroinvertebrate a) log-transformed mean abundance (Slope = 0.06, SE = 0.07, t = 0.9, p = 0.38) and b) mean species richness (Slope = 0.02, SE = 0.007, t = 2.8, p = 0.01) to mean values of CO_2_ flux (g CO_2_ m^-2^ hour^-1^). Data point shape indicates the study island and data point color indicates the study beach. Data points are mean values for a site visit from 2016 to 2018 (year not graphically displayed and sites visited 1 to 3 times).

CO_2_ flux measurements made along the high tide strandline were also highly variable across sites and surveys ranging from 0.05 to 1.2 g CO_2_ m^-2^ hour^-1^ (Table 1). Highest values of CO_2_ flux (> 0.3 CO_2_ m^-2^ hour^-1^) were observed at the three beaches with the highest wrack standing stock (>3 m^2^ m^-1^) mentioned above. We found a strong linear relationship between our *in-situ* measures of beach CO_2_ flux and wrack standing stock (Fig 3, Slope = 0.06, SE = 0.009, t = 7.09, p < 0.0001). We also found a significant relationship for wrack standing stock with species richness (Fig 4A, Slope = 0.02, SE = 0.007, t = 2.8, p = 0.01), but not with log-transformed mean abundance (Fig 4B, Slope = 0.06, SE = 0.07, t = 0.9, p = 0.38) of wrack-associated macroinvertebrates. The linear mixed effects model evaluating several potential drivers of CO_2_ flux explained significantly more variation in CO_2_ flux than the null model (ANOVA, Likelihood Ratio = 37.34, p < 0.0001) and identified marine wrack cover as the primary driver of CO_2_ fluxes (Table 2, t-value = 4.53, p = 0.0001).

**Table 2:** Linear mixed effects model results. Parameter estimate, 95% confidence intervals (CI), and p-value are given with significant predictors denoted in bold.

Predictor	t	Estimate	CI	p
(Intercept)	1.9	0.28	0.0001 - 0.569	0.074
Air temperature	−1.6	−0.01	−0.23 - 0.002	0.116
Upper beach width	1.2	0.003	−.002 - 0.008	0.244
**Wrack cover**	**4.5**	**0.06**	**0.035 - 0.085**	**0.0001**
Macroinvertebrate species richness	0.2	0.001	−.013 - 0.016	0.850
Macroinvertebrate abundance	−0.9	0	0 − 0	0.373

**Fig 5:**
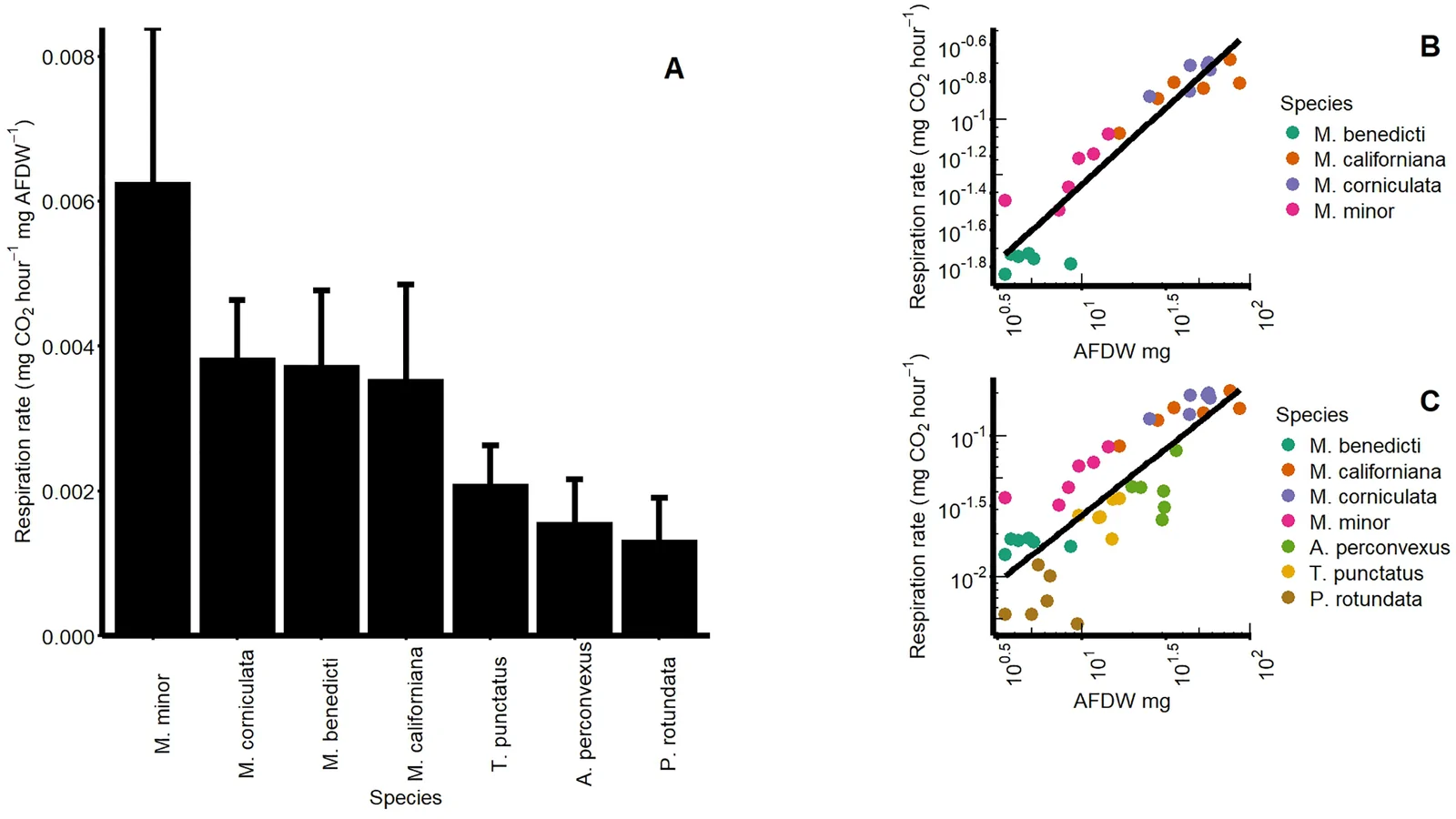
Macroinvertebrate respiration. a) Mean values of (n = 6) biomass-normalized species-specific respiration rates (mg CO_2_ hour^-1^ mg^-1^ AFDW) for adults of seven common and abundant wrack-consuming macroinvertebrates, b) log-transformed respiration rate as a function of individual log-transformed AFDW (mg) for *Megalorchestia* spp. (r^2^ = 0.88, p < 0.0001, slope = 0.832) and c) for all seven species (r^2^ = 0.66, p < 0.0001, slope = 0.947).

Laboratory measurements of respiration rate at rest under standard experimental conditions for seven upper intertidal beach macroinvertebrate detritivore species (n = 6 adults per species) varied more than four-fold among species and scaled with body size. The four talitrid amphipod species yielded values of 0.0038 ± 0.0008 (SD) mg CO_2_ hour^-1^ mg^-1^ AFDW for *Megalorchestia corniculata*, 0.0036 ± 0.001 mg CO_2_ hour^-1^ mg^-1^ AFDW for *M. californiana*, 0.0063 ± 0.002 mg CO_2_ hour^-1^ mg ^−1^ AFDW for *M. minor* and 0.0037 ± 0.001 mg CO_2_ hour^-1^ mg^-1^ AFDW for *M. benedicti* (Fig 5A). Talitrid amphipods, *Megalorchestia* spp., are typically the most abundant wrack-associated macroinvertebrate across southern California, and two or more species coexist on many beaches [46]. We found that respiration rates are a strong function of body size at the genus level for *Megalorchestia* (Fig 5B, R^2^ = 0.88, p < 0.0001). *Megalorchestia spp.* CO_2_ flux (mg CO_2_ hour^-1^ mg^-1^ AFDW) can be calculated as:


$$\begin{array}{c} y=\;-\text{2}.\text{1}\text{8}\text{6}+\text{0}.\text{8}\text{3}\text{2}x \end{array}$$

where y is log-transformed CO_2_ flux and x is log-transformed individual ash-free dry weight (mg). For the two isopod species, values were similar at 0.0016 ± 0.0006 mg CO_2_ hour^-1^ mg^-1^ AFDW for *Alloniscus perconvexus* and 0.0021 ± 0.0005 mg CO_2_ hour^-1^ mg^-1^ AFDW for *Tylos punctatus* (Fig 5A). The tenebrionid beetle *Phaleria rotundata* had the lowest rate at 0.0013 ± 0.0006 mg CO_2_ hour^-1^ mg^-1^ AFDW (Fig 5A). Across the seven species tested here, respiration is similarly a strong function of body size (Fig 5C, R^2^ = 0.66, p < 0.0001) and can be calculated with the same approach using:


$$\begin{array}{c} y=\;-\text{2}.\text{5}\text{1}\text{1}+\text{0}.\text{9}\text{4}\text{7}x \end{array}$$

**Fig 6:**
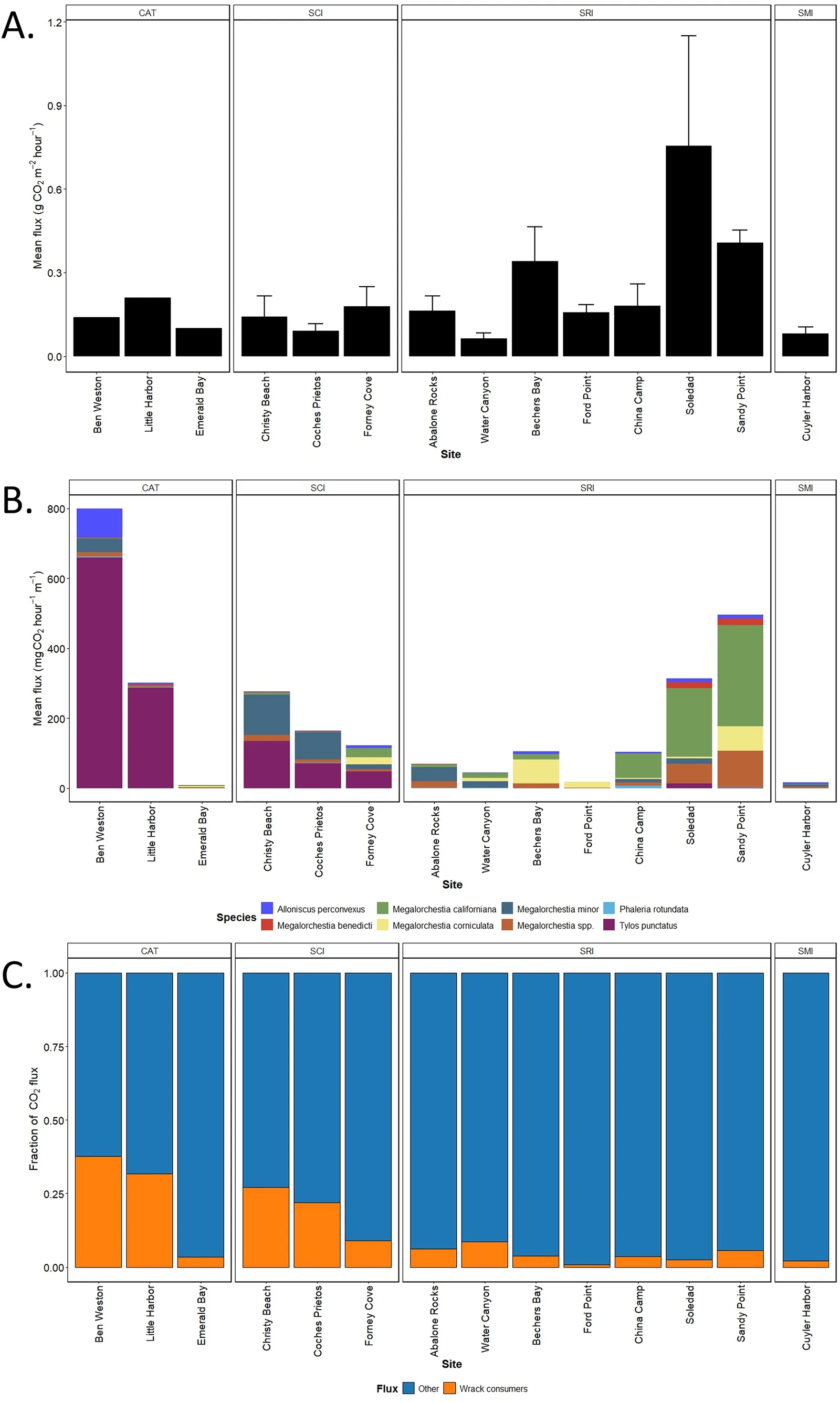
CO_2_ flux and estimated consumer contributions. A) Mean (± standard deviation) values of sediment CO_2_ flux (g CO_2_ m^-2^ hour^-1^) for each study beach. B) Beach-scale consumer respiration rates (mg CO_2_ hour^-1^ m^-1^) of the seven species of wrack-consuming macroinvertebrates derived from their laboratory-measured respiration rates and field measurements of AFDW biomass per meter of shoreline. C) Estimates of the proportion of beach-scale CO_2_ flux that can be attributed to the seven species of wrack-consuming macroinvertebrates at our study sites. For panels A-C, data presented are mean values for all site visits (n = 1 to 3) from 2016 to 2018 and sites are ordered from east to west.

We used the laboratory-measured respiration rates and field measurements of population biomass for these seven species of detritivores to estimate their cumulative contribution to the total CO_2_ flux rates for each beach site by island (Fig 6A). Estimated mean values of beach-scale respiration rates for the assemblage of these detritivore consumers ranged from 0.003 ± 0.003 (1.6 ± 2.8 mg AFDW biomass m^-1^ *Phaleria rotundata*) to 659.1 ± 431.7 (308,335.6 ± 191,219.0 mg AFDW biomass m^-1^ *Tylos punctatus*) mg CO_2_ hour^-1^ m^-1^ shoreline and did not follow a geographic pattern across the four islands (Fig 6B). This flux was dominated by contributions from the abundant species, the isopods *T. punctatus* and *A. perconvexus* and the talitrid amphipod *M. minor* on beaches located on islands to the south and east (Santa Catalina Island and Santa Cruz Island). On the beaches of islands to the west (Santa Rosa Island and San Miguel Island), consumer respiration was dominated by talitrid amphipods, predominantly *M. californiana*, *M. corniculata*, and juvenile *Megalorchestia spp*. At the beach scale, we estimate the consumer CO_2_ flux ranged from 0.8 ± 0.6 mg CO_2_ hour^-1^ m^-1^ shoreline at Ford Point Beach on Santa Rosa Island to 798.6 ± 437.0 mg CO_2_ hour^-1^ m^-1^ shoreline at Ben Weston Beach on Santa Catalina Island (Fig 6B). Our estimates of the fraction of beach ecosystem-scale CO_2_ flux that could be attributed to the guild of abundant wrack-associated macroinvertebrates showed high across site variability, ranging from 0.9% to 37.7% with an overall mean of 11.8% (Fig 6C). The greatest estimates for consumer fractions (>20%) were found on two Santa Catalina Island and two Santa Cruz Island beaches where the isopod *Tylos punctatus* was the dominant wrack-associated macroinvertebrate (Fig 6B). In fact, the consumer contribution to beach-scale CO_2_ flux decreased with greater beach hopper (*Megalorchestia spp.*) proportional abundance within the full invertebrate community and increased with greater isopod (*Tylos puncatus* and *Alloniscus perconvexus*) proportional abundance (S3 Figure in S1 File).

## Discussion

As basal resources for the sandy beach food web, spatially patchy and temporally dynamic marine macrophyte wrack inputs elicit strong responses from the intertidal macroinvertebrate community and enhance ecosystem functioning and respiration rates. Our findings suggest that high tide strandlines on beaches with high wrack cover function as biogeochemical hotspots with very high CO_2_ production compared to the surrounding habitat [36], similar to ant hills on the forest floor [52,53], termite mounds in savanna soils [54], and dung pats occupied by beetles [55]. The space-for-time approach employed here demonstrated that strong coupling between wrack inputs, macroinvertebrate populations, and CO_2_ flux occurs irrespective of beach site. The inference of this result is that an increase or decrease in wrack inputs over time would likely have a positive or negative impact, respectively, on the invertebrates and CO_2_ flux. The standing stock of marine macrophyte wrack varied greatly across our study beaches and was a strong predictor of sediment CO_2_ flux at the high tide strandline. Wrack was the dominant driver of CO_2_ flux while consumers likely modulated this relationship. Determining the independent contribution of consumers through experimental isolation, therefore, would be a strong avenue for future research and overcome limitations associated with inferring their role from observational data.

Wrack processing and subsequent nutrient recycling, important ecosystem functions on sandy beaches, are primarily driven by consumption activities of organisms ranging from microbes [56,57] to macroinvertebrate detritivores [28,29,34]. Here, wrack standing stock was a strong driver of the abundance and species richness for upper intertidal macroinvertebrates [58], many of which are detritivores that feed on wrack [28]. The increase in sediment CO_2_ flux with macroinvertebrate abundance and species richness is indicative of a biodiversity-supported biogeochemical functional response to wrack subsidies. Wrack detritivores functionally operate as the shredders of the beach, analogous to those in streams [59,60]. Increased detritivore abundances can enhance ecosystem CO_2_ flux from the consumption and subsequent respiration of detrital organic matter [61,62]. Through shredding activity, which increases detrital surface area, they can also enhance the availability of wrack particles to meiofauna and microbes, promoting greater total decomposition and respiration of organic matter [60,63–65]. Similar ecological and biological phenomena have been observed where subsidies provide patchy, high-energy inputs into low productivity environments [14,66,67].

The range of mean CO_2_ flux rates observed in our study (n = 34 observations, 0.05 to 1.18 g CO_2_ m^-2^ h^-1^) were higher than the few examples available from other sandy beaches for *in situ* (i.e., no experimental manipulation) sediment CO_2_ flux rates over bare sand (Table 3). In fact, our flux rates were comparable to a number of studies which took measurements directly over wrack patches. Measurements taken directly over wrack would likely overestimate beach-scale fluxes compared to our measurements of wrack-influenced sediment along the high tide strandline. For example, beaches in southwestern Australia had measured values of 0.16 g CO_2_ m^-2^ h^-1^ on bare sand and 1.05 g CO_2_ m^-2^ h^-1^ directly through wrack [36], both of which were similar to our range of flux measurements in bare sand. Experimental studies that measured the sediment CO_2_ flux rate directly through wrack patches placed on the beach ranged from 0.03 to 0.71 g CO_2_ m^-2^ h^-1^ (Table 3) [21,37,39]. There are extensive, *in situ* ecosystem metabolism measurements for other soft-sediment intertidal habitats, including seagrass meadows, salt marshes, and mangrove forests, both during submersion and emersion, and often in the context of blue carbon budgeting [68–70]. Sediment CO_2_ flux rates from our study were generally of the same magnitude observed for other coastal ecosystems with relatively high amounts of detritus including salt marsh (0.001–0.15 g m^-2^ h^-1^) [71–74], tidal flat (0.04–0.44 g m^-2^ h^-1^) [75,76], and mangrove (0.14–0.39 g m^-2^ h^-1^) [68,77,78], ecosystems measured during emersion. Environmental variables, such as dry upper beach width, sediment moisture and air temperature were not significant factors in our results, but may be important factors in other beach or coastal ecosystems.

**Table 3:** The range of mean values of CO_2_ flux on sandy beaches from the literature. Flux rate measurements are denoted as from sand, wrack, and/or experimental manipulations.

Location	Description	Substrate	Mean CO_2_ flux (g CO_2_ m^-2^ h^-1^)	Reference
Channel Islands, California, USA	*In situ* measurements through bare sand, 14 beaches, Aug – Nov, 2016–2018. Wrack dominated by *Macrocystis pyrifera, Phyllospadix torreyi*	Sand	0.230	This Study
Port Foster, Deception Island	*In situ* measurements through bare sand, 12 beaches, Feb 2016; Feb and Mar 2017 with wrack dominated by *Palmaria decipiens;* Feb 2016 experiment measured through *Palmaria decipiens* and adjacent bare sand	Sand	0.053	[40]
		Wrack (Experiment)	0.271	
		Sand (Experiment)	0.022	
Galicia, Spain	Experiment, one beach, Jun and Jul 2014 with measurements from placed *Saccorhiza polyschides* treatments and adjacent bare sand	Sand	0.007	[39]
		Fresh Wrack	0.033	
		Aged Wrack	0.119	
Galicia, Spain	Experiment, two beaches, Mar 2015 with measurements from placed *Saccorhiza polyschides, Cystoseira baccata, Sargassum muticum, Undaria pinnatifida* and adjacent bare sand	Sand	0.016	[21]
		Wrack	0.610	
Galicia, Spain	Experiment, one beach, Jun 2011 with measurements from placed *Laminaria spp.* patches and adjacent bare sand	Sand	0.051	[37]
		Wrack	0.707	
Southwest Australia	*In situ* Measurements, 16 beaches, Nov 2006. Wrack dominated by *Posidonia australis, Sargassum* spp.	Sand	0.160	[36]
		Wrack	1.050	
Wimereux, France	*In situ,* One beach, Apr 2000 – May 2003 occasionally high concentrations of microalgae *Euglena spp., Phaeocystis spp.*	Sand	0.002	[79]
Wimereux, France	*In situ,* One beach, Oct 2000	Sand	0.002	[80]

Macrofaunal respiration in coastal benthic environments is estimated to contribute up to 30% of total community respiration across subtidal habitat types and 12% with respect to soft-sediment habitats alone [81]. CO_2_ flux on beaches with sparse populations of consumers is generally low and associated with microbial activity [79]. A macrofaunal exclusion experiment on sandy beaches in Portugal estimated a 20% contribution of macroinvertebrates to CO_2_ flux [37]. Our finding that respiration by the most common and abundant species on our study beaches, wrack detritivores, comprised an average of ~11.8% of beach sediment CO_2_ flux, a lower value compared to other studies, suggests a higher contribution of microbial respiration than found in other studies. Importantly, our estimates of beach-scale sediment CO_2_ flux were based on direct respiration by the consumer species. Therefore, the role of consumers estimated here is likely a conservative estimate as it does not account for the indirect effects of these species on other organisms or processes on the beach which could affect the net sediment CO_2_ flux. The scaling of individual respiration rates to body mass within *Megalorchestia* approached the 3/4-power law of metabolism, a physiological relationship attributed to constraints of internal resource use and distribution across highly conserved body plans [82]. In contrast, evaluating the full suite of seven macroinvertebrate species yielded a scaling exponent near 1.0, likely resulting from aggregation of distinct taxonomic groups with differing baseline metabolic efficiencies. This dynamic has been noted when modeling broad, phylogenetically diverse benthic assemblages where interspecific variations elevate the overall community-level scaling (Mahaut et al., 1995). Importantly, there is significant variation in metabolic scaling with body size driven by differences in surface area and mass/volume ratio within and across species, such that power scaling is unlikely universal [83,84].

Burrowing invertebrates are important contributors to CO_2_ fluxes across intertidal ecosystems due to bioturbation-driven enhancement of organic matter remineralization [85]. The presence of fiddler crab (*Uca* spp.) burrows plus respiration from occupied burrows greatly increased the CO_2_ flux from intertidal mangrove sediments [77]. Similarly, in salt marshes, crab presence increased the flux of CO_2_ from intertidal sediments [70,86]. Evidence from other ecosystems echoes the important role of macroinvertebrates with respect to greenhouse gas fluxes. Litter decomposers from forest ecosystems increased soil CO_2_ flux by 18% [87] and ant mounds contributed 7% of total marsh wetland CO_2_ flux [88]. In urban wetlands, each doubling of invertebrate densities increased methane and carbon dioxide fluxes by 42% and 15%, respectively [89]. Benthic macrofauna increased methane fluxes by 8-fold and account for 9.5% of total Baltic Sea methane emissions [90]. In sandy coastal sediments, the presence of a burrowing polychaeta resulted in a 50% increase in CO_2_ release [91]. In Antarctica, the dominant soil invertebrate responsible for 2–7% of soil CO_2_ flux is rapidly declining in abundance due to climate warming and the implications of losing this dominant species with respect to ecosystem functioning in such a low-diversity system may be significant [92,93]. As with other ecosystems, the majority of the sediment CO_2_ flux from sandy beaches is likely associated with microbial respiration, although meiofauna may be significant contributors [30,37,94]. Still, respiration by detritivores is an important contributor to the total overall flux and their feeding and excretion may facilitate microbial respiration and nutrient recycling [37], greatly adding to their role in ecosystem functioning and decomposition.

Consumer respiration rates scaled to the beach level indicated that the dominant macroinvertebrate contributor to ecosystem-scale CO_2_ flux varied among islands and sites. Our results indicated that the respiration of upper intertidal macroinvertebrate wrack consumers of beaches on average likely contributes 11.8% of the ecosystem-level CO_2_ flux. However, their feeding and burrowing activities likely contribute more through shredding and bioturbation-driven burial of wrack and oxygenation of buried decomposing wrack for microbial activity [95,96]. The much higher respiration rates of individuals of the two largest talitrid species matches well with their higher rates of wrack consumption [34]. The key functional role of these two talitrid species relative to the other common wrack detritivores is clear and highlights the importance of considering species identity and functional roles in conservation planning [33,93,97]. The beaches with the highest consumer contribution to CO_2_ flux rates (27% to 37%) were dominated by the isopod *Tylos punctatus* and had low to moderate wrack cover. Oniscid isopods consume wrack at lower rates than talitrid amphipods [34]. This reduces the rate of shredding and potentially the availability of the wrack organic matter to microbial activity. Thus, on beaches where talitrids dominate they may facilitate greater connectivity between wrack inputs and microbial decomposers via shredding and ‘sloppy feeding’ compared to isopods, again demonstrating how species identity can greatly influence ecosystem functioning [34,98,99]. Organisms which dominate in terms of abundance or biomass do not necessarily dominate rates of resource use and respiration which leaves open questions regarding perceived disparities between energy-use dynamics, community structure, and niche theory [100,101].

A beach ecosystem-scale approach allows for more informed and better constrained estimates of ecosystem-scale CO_2_ flux that can be used to more accurately identify and assess the effects of climate change on ecosystem processes [43,102]. The potential contribution of nearshore macrophytes to blue carbon is highly uncertain as the fate of this material, including the processes surrounding its breakdown, consumption, and/or decomposition, is largely unknown [103–106]. Our findings inform the carbon budgeting of coastal ecosystems, including evaluation of the fate of blue carbon derived from macroalgal and seagrass primary production [107–110], and indicate that in addition to significant direct consumption by detritivores [28,34], there are also high rates of remineralization to CO_2_ through decomposition. While macroalgal wrack decomposes much faster than seagrass wrack [111,112], the relative contribution of each wrack type to beach CO_2_ flux in relation to their respective input rates to beaches has yet to be explored.

Ecosystem functioning is tightly coupled to subsidies and the incorporation of subsidies into the food web in many systems [38,113]. Because of the dynamic nature of beaches, we expect fluctuations in functioning to coincide with spatial and temporal variation in habitat condition, wrack inputs, and macroinvertebrate populations [114]. As we show here this is especially true of the upper intertidal zone, the area from the high tide strandline to the upper beach limit (i.e., cliff or dune base), where deposition and retention of wrack subsidies predominantly occurs. Here, accumulations of wrack act as hotspots that affect community structure by influencing biodiversity, abundance, and biomass of macroinvertebrates and driving many ecological processes such as the provisioning of prey for invertebrate and vertebrate predators, including shorebirds, enhancing secondary productivity, and promoting nutrient regeneration in this relatively narrow strip of intertidal habitat [27,31,32,35,58,115].

Like many ecotones with high relative levels of ecosystem functioning, this biogeochemically and ecologically active zone of the sandy beach ecosystem is critically imperiled in the face of rising sea levels and anthropogenic development, including coastal squeeze [116–119]. The upper intertidal zone of beaches in southern California is already impacted by widespread coastal management practices (e.g., armoring, grooming) [119–121] and is particularly vulnerable to impacts from climate change [117,118]. Projections for California predict the loss of the upper intertidal beach zone by the end of the century with many bluff-backed and armored beaches having already reached a tipping point [116,118,122]. Food web dynamics of key donor ecosystems for beaches like rocky reefs may also be altered by warming temperatures, increased nutrient loading, growing intensity of storms, and shifting resource availability [123–126] profoundly affecting subsidies that drive beach function and biodiversity. The strong coupling of subsidies, intertidal consumers, and biogeochemical processes in this ecotonal hotspot demonstrates the vulnerability of key ecosystem processes on sandy beaches to rising sea levels and coastal development. Ecological hotspots which harbor biodiversity and provide some of the highest levels of ecosystem functions need to be identified and targeted for conservation and restoration [127]. This need is particularly urgent for ecotones like beaches whose role in coastal ecosystem functioning is just beginning to be recognized, yet are already losing ground to pressures from the land and the sea.

## Supporting information

S1 File The Supporting Information file contains the following five elements. S1 Table: Mean values of survey data. Mean values of survey data from each sampling event. Data collection for each is described in the methods section of the manuscript. S2 Table: Invertebrate allometric biomass equations. Allometric equations used to convert invertebrate biomass from wet weight to dry weight and ash-free dry weight (AFDW). S1 Figure: CO_2_ flux based on beach location. Mean ± SE CO_2_ flux (g CO_2_ m^-2^ hour^-1^) at different locations across the beach face including the dry upper beach, the 24-hour high tide strandline, the 12-hour high tide strandline and the saturated lower beach. S2 Figure: Species-specific beach-scale respiration estimates. The estimated total respiration rate at the beach scale (per meter of shoreline) for the focal consumer species by survey. S3 Figure: CO_2_ flux based on percent of abundance for beach hoppers and isopods. The consumer contribution to beach-scale sediment CO_2_ flux in relation to the percent of total invertebrate abundance comprised by beach hoppers (*Megalorchestia spp.*) or isopods (*Alloniscus perconvexus* or *Tylos punctatus*). Consumer contribution to beach-scale CO_2_ flux decreased if beach hoppers were more abundant (weighted linear regression r^2^ = 0.65, p < 0.0001) but increased if isopods were more abundant (weighted linear regression r^2^ = 0.52, p < 0.0001).
(DOCX)
