# Supplementary material for: Cross-ecosystem subsidies drive CO2 flux rates in a coastal ecotone
Source: PLoS One. 2026 Sep 11;21(9):e0357828. doi: 10.1371/journal.pone.0357828 (PMC13567790; doi:10.1371/journal.pone.0357828)
Supplement: Supplementary file 1 [file pone.0357828.s001.docx]

**Supplementary Tables**

**S1 Table:** **Mean values of survey data.** Mean values of survey data from each sampling event. Data collection for each is described in the methods section of the manuscript.

| **Island** | **Site** | **Year** | **Air temperature (˚C)** | **Mean dry upper beach width (m)** | **Mean HTS slope (˚)** | | **Beach length (m)** | **Beach orientation (˚)** | **Marine wrack cover (m^2^ m^-1^)** | **CO_2_ Flux (g CO_2_ m^-2^ hour^-1^)** | **Total upper beach invertebrate richness** | **Mean upper beach invertebrate abundance (ind. m^-1^)** |
| --- | --- | --- | --- | --- | --- | --- | --- | --- | --- | --- | --- | --- |
| CAT | Ben Weston | 2018 | 26.0 | 28.8 | | 6.0 | 0.95 | 14 | 2.43 | 0.14 | 17 | 112747.2 |
|  | Little Harbor | 2018 | 26.0 | 8.5 | | -0.9 | 1.93 | 324 | 1.64 | 0.21 | 9 | 33655.9 |
|  | Emerald Bay | 2018 | 22.0 | 6.0 | | 0.8 | 1.15 | 322 | 0.43 | 0.10 | 4 | 513.5 |
| SCI | Coches | 2016 | 22.3 | 13.2 | | 1.1 | 1.93 | 324 | 0.49 | 0.08 | 5 | 20155.3 |
|  |  | 2017 | 20.9 | 17.7 | | 5.5 | 1.15 | 322 | 0.60 | 0.08 | 7 | 7355.1 |
|  |  | 2018 | 14.4 | 12.2 | | 5.8 | 0.26 | 117 | 0.32 | 0.12 | 8 | 17748.9 |
|  | Christy's | 2016 | 17.5 | 4.9 | | 3.6 | 1.15 | 322 | 0.28 | 0.08 | 7 | 27565.6 |
|  |  | 2017 | 19.2 | 10.4 | | 5.5 | 0.95 | 14 | 0.25 | 0.13 | 8 | 39181.7 |
|  |  | 2018 | 18.9 | 13.2 | | 1.6 | 1.25 | 232 | 0.99 | 0.22 | 9 | 43867.2 |
|  | Forney's | 2016 | 19.3 | 5.7 | | 2.5 | 1.25 | 232 | 0.31 | 0.15 | 12 | 7180.2 |
|  |  | 2017 | 22.8 | 16.5 | | 5.2 | 1.93 | 324 | 0.95 | 0.13 | 7 | 14120.2 |
|  |  | 2018 | 14.7 | 17.4 | | 5.1 | 0.36 | 56 | 0.55 | 0.26 | 7 | 15482.6 |
| SRI | Abalone Rocks | 2016 | 18.4 | 5.2 | | 6.8 | 0.26 | 117 | 1.42 | 0.20 | 14 | 6152.3 |
|  |  | 2017 | 18.0 | 13.8 | | 2.6 | 0.26 | 117 | 2.03 | 0.13 | 14 | 5604.8 |
|  | Water Canyon | 2016 | 25.4 | 9.1 | | 2.3 | 0.26 | 134 | 0.43 | 0.06 | 8 | 2165.8 |
|  |  | 2017 | 17.9 | 10.4 | | 4.1 | 0.26 | 134 | 0.03 | 0.05 | 8 | 1538.1 |
|  |  | 2018 | 22.8 | 13.2 | | 7.3 | 0.25 | 229 | 0.34 | 0.09 | 11 | 2141.6 |
|  | Bechers | 2016 | 23.9 | 7.5 | | 12.2 | 0.36 | 56 | 1.65 | 0.34 | 9 | 12467.8 |
|  |  | 2017 | 15.4 | 12.4 | | 2.9 | 0.36 | 56 | 4.18 | 0.22 | 11 | 12673.8 |
|  |  | 2018 | 13.0 | 12.9 | | 3.5 | 0.78 | 167 | 3.21 | 0.46 | 9 | 11625.9 |
|  | Ford Pt | 2016 | 20.1 | 2.9 | | 2.9 | 0.39 | 76 | 0.20 | 0.13 | 2 | 1748.6 |
|  |  | 2017 | 23.5 | 8.9 | | 4.1 | 0.39 | 76 | 0.16 | 0.17 | 2 | 338.7 |
|  |  | 2018 | 23.0 | 4.5 | | 4.6 | 0.26 | 134 | 0.35 | 0.18 | 1 | 445.6 |
|  | China Camp | 2016 | 30.5 | 29.5 | | 6.0 | 2.25 | 49 | 2.16 | 0.23 | 10 | 6317.8 |
|  |  | 2017 | 18.3 | 46.3 | | 2.3 | 2.25 | 49 | 0.69 | 0.23 | 8 | 6096.3 |
|  |  | 2018 | 19.3 | 28.6 | | 6.4 | 1.9 | 286 | 2.03 | 0.09 | 15 | 9648.6 |
|  | Soledad | 2016 | 29.2 | 26.6 | | 13.9 | 1.9 | 286 | 11.99 | 0.41 | 16 | 57288.0 |
|  |  | 2017 | 19.9 | 47.2 | | 20.2 | 1.9 | 286 | 9.59 | 1.18 | 20 | 22545.2 |
|  |  | 2018 | 19.1 | 20.1 | | 7.3 | 0.26 | 279 | 6.39 | 0.67 | 18 | 30784.3 |
|  | Sandy Pt | 2016 | 16.9 | 8.9 | | 7.0 | 0.78 | 167 | 4.18 | 0.38 | 23 | 66524.1 |
|  |  | 2017 | 19.0 | 41.7 | | 4.0 | 0.78 | 167 | 4.84 | 0.38 | 18 | 28885.9 |
|  |  | 2018 | 17.1 | 14.5 | | 2.8 | 0.31 | 61 | 4.64 | 0.46 | 17 | 25777.9 |
| SMI | Cuyler's | 2017 | 16.7 | 6.8 | | 8.2 | 1.25 | 232 | 0.11 | 0.07 | 6 | 1897.1 |
|  |  | 2018 | 17.7 | 26.3 | | 7.1 | 2.25 | 49 | 0.07 | 0.10 | - | - |

**S2 Table: Invertebrate allometric biomass equations.** Allometric equations used to convert invertebrate biomass from wet weight to dry weight and ash-free dry weight (AFDW).

| **Species** | **Wet to dry mass conversion factor (± SD)** | **Dry to ash-free dry mass conversion factor (± SD)** |
| --- | --- | --- |
| Megalorchestia benedicti | 0.28 ± 0.006 | 0.73 ± 0.05 |
| Megalorchestia minor | 0.27 ± 0.005 | 0.71 ± 0.08 |
| Megalorchestia corniculata | 0.28 ± 0.003 | 0.66 ± 0.02 |
| Megalorchestia californiana | 0.26 ± 0.006 | 0.67 ± 0.07 |
| Alloniscus perconvexus | 0.29 ± 0.006 | 0.57 ± 0.02 |
| Tylos Punctatus | 0.28 ± 0.004 | 0.50 ± 0.04 |
| Phaleria rotundata | 0.21 ± 0.006 | 0.92 ± 0.06 |

**Supplementary Figures**

**S1 Figure:** **CO_2_ flux based on beach location.** Mean ± SE CO_2_ flux (g CO_2_ m^-2^ hour^-1^) at different locations across the beach face including the dry upper beach, the 24-hour high tide strandline, the 12-hour high tide strandline and the saturated lower beach

| Location on Beach | CO2 Flux (g CO2 m-2 hr-1) |
| --- | --- |
| Dry Upper beach | 0.15 |
| 24-hr HTL | 1.7833 |
| 12-hr HTL | 0.2866 |
| Saturated Lower Beach | 0.05 |

**S2 Figure:** **Species-specific beach-scale respiration estimates.** The estimated total respiration rate at the beach scale (per meter of shoreline) for the focal consumer species by survey.

**S3 Figure:** **CO_2_ flux based on percent of abundance for beach hoppers and isopods.** The consumer contribution to beach-scale sediment CO_2_ flux in relation to the percent of total invertebrate abundance comprised by beach hoppers (*Megalorchestia spp.*) or isopods (*Alloniscus perconvexus* or *Tylos punctatus*). Consumer contribution to beach-scale CO_2_ flux decreased where beach hoppers were more abundant (weighted linear regression r^2^ = 0.64, p < 0.0001) but increased where isopods were more abundant (weighted linear regression r^2^ = 0.51, p < 0.0001)
